# The detection and significance of *EGFR* and *BRAF* in cell-free DNA of peripheral blood in NSCLC

**DOI:** 10.18632/oncotarget.17937

**Published:** 2017-05-17

**Authors:** Yang Yang, Xiaoyan Shen, Rutian Li, Jie Shen, Hang Zhang, Lixia Yu, Baorui Liu, Lifeng Wang

**Affiliations:** ^1^ The Comprehensive Cancer Center of Drum Tower Hospital, Nanjing University Medical School and Clinical Cancer Institute of Nanjing University, Nanjing 210008, PR China; ^2^ Nanjing Xianlin Drum Tower Hospital, Nanjing 210046, PR China

**Keywords:** NSCLC, cfDNA, CastPCR, driver mutation, EGFR

## Abstract

**Objective:**

Although driver mutation status is crucial to targeted therapy decision-making in non-small cell lung cancer (NSCLC), due to unavailable or inadequate biopsies, there are still many patients with unknown mutation status. A promising way to solve this problem is liquid biopsy, such as cell-free DNA (cfDNA) in peripheral blood. Additionally, due to the little amount of cfDNA, detecting methods with high sensitivity, specificity and economy are required in clinical practice. Here, we explored the feasibility of Competitive Allele-Specific TaqMan^®^ PCR (CastPCR) detecting driver mutations in cfDNA from plasma in lung adenocarcinoma patients.

**Results:**

Sensitivity, specificity, concordance, PPV and NPV of CastPCR detecting EGFR mutations in cfDNA was 56.4% (31/55), 94.2% (49/52), 74.8% (80/107), 91.2% (31/34) and 67.1% (49/73), respectively. Notably, specificity and PPV for p.T790M both reached 100.0%. For BRAF detection, it was 28.6% (2/7), 93.0% (93/100), 88.8% (95/107), 22.2% (2/9) and 94.9% (93/98), respectively.

**Materials and Methods:**

Plasma specimens of 107 lung adenocarcinoma patients and their matched tumor formalin fixed paraffin embedded (FFPE) samples were analyzed. CastPCR was used to detect EGFR (c.2235_2249del, c.2236_2250del, c.2369C>T p.T790M, c.2573T>G p.L858R) and BRAF (c.1406G>C p.G469A, c.1799T>A p.V600E, c.1781A>G p.D594G) mutations. Mutation results of tumor tissue was set as gold standard, and the sensitivity, specificity, concordance, positive predictive value (PPV) and negative predictive value (NPV) were calculated for each mutation.

**Conclusions:**

For patients whose tumor tissue is unavailable or inadequate, EGFR mutation detection in cfDNA with CastPCR could be first choice. Mutation positive results may provide reference for further clinical medication. While negative results indicate that detection in tissue should be considered as the following step. In this way, tumor tissue could be economized to the maximum extent and the risk of repeated percutaneous transthoracic lung biopsy could also be lowered to the maximum extent. For BRAF detection in cfDNA, CastPCR is a specific method while the sensitivity needs further exploration.

## INTRODUCTION

Thanks to *EGFR* tyrosine kinase inhibitors (*EGFR*-TKIs), the treatment pattern of metastatic NSCLC has been revolutionized. Patients' progress free survival, overall response rate and quality of life have been ameliorated significantly [1–7]. However, in clinical practice, detection of driver mutations in tumor tissue is often not enough. First, sufficient tumor tissue is not readily available. For instance, only 35.9% (437/1217) and 20.3% (297/1466) of the advanced NSCLC patients had biopsied tissue that was suitable for testing in IPASS study [1] and INTEREST study [8], respectively. Second, due to heterogeneity, landscape of molecular information can't be covered with just one or two spots of biopsy [9]. Third, since tumor is evolving under drug therapy, dynamic and real-time monitoring of driver mutations during treatment is required. However, repeated biopsy is not practically feasible in clinical work for its invasiveness and risk. Fortunately, liquid biopsy such as cfDNA from plasma [10–12] has come into the picture. cfDNA originates from apoptosis, necrosis, phagocytosis, oncosis and active secretion of cells [13]. Studies have demonstrated that in cancer patients, cfDNA contains representation of the entire tumor genome [14, 15]. The mutation load was reported higher than 25.0% in one third of mutant plasma samples [13]. There have been multiple publications on cfDNA mutation detection in the last two decades [16–25]. According to Committee for Medicinal Products for Human Use (CHMP) of the European Medicines Agency (EMA), to predict therapeutic response of Iressa® (gefitinib), the use of circulating tumor DNA obtained from a blood sample has been allowed to assess *EGFR* mutation status in patients where a tumor sample is not an option[13]. Additionally, cfDNA was shown to be useful in dynamic monitoring of acquired resistance and prediction of relapse in various cancers, including NSCLC [26–30]. In clinical practice, methods with high sensitivity, specificity, convenience and economy are required. Development of molecular detection methods such as qPCR based methods, would allow for highly specific analysis of cfDNA [31]. Here, we evaluated the use of CastPCR detecting *EGFR* mutations (c.2235_2249del, c.2236_2250del, p.T790M, and p.L858R) in cfDNA form plasma in adenocarcinoma patients.

Reported incidence of BRAF mutations ranges from 0.5–9.0% in NSCLC [32]. Phase II clinical trial NCT01336634 has made its results public: *BRAF* targeted inhibitor dabrafenib has shown clinical activity in *BRAF* p.V600E-positive metastatic NSCLC. It suggested that for patients with limited therapeutic options, dabrafenib could represent a treatment option [33]. Detection of *BRAF* mutations in cfDNA hasn't been reported in NSCLC, thus we also evaluated CastPCR technology in detecting *BRAF* mutations (p.G469A, p.V600E and p.D594G).

## RESULTS

### *EGFR* mutations

Of the 107 plasma specimens, 31.8% (34/107) cases were *EGFR* mutated, including 76.5% (26/34) sensitive mutations (c.2235_2249del/c.2236_2250del or/and p.L858R) and 26.5% (9/34) resistant mutations (p.T790M). A 19 + 20 double mutation (two different mutations found simultaneously in one patient) was found in one patient (0.9%, 1/107). In FFPE samples, 51.4% (55/107) cases were *EGFR* mutated: 72.3% (40/55) patients harbored sensitive mutations and 36.4%, (20/55) harbored resistant mutations (Tables 1, 2). In addition, 5.6% (6/107) mutation-positive patients were double-mutated: 3 with c.2235_2249del/c.2236_2250del + p.T790M, 1 with c.2235_2249del/c.2236_2250del + p.L858R and 2 with p.T790M + p.L858R. Sensitivity, specificity, concordance, PPV and NPV of CastPCR detecting *EGFR* mutations in cfDNA are listed in Tables 3, 4.

**Table 1:** *EGFR* sensitive mutations (c.2235_2249del/c.2236_2250del or/and p.L858R) in cfDNA and FFPE samples

FFPE	Mutation (−)	Mutation (+)	Total
cfDNA			
Mutation (+)	3	23	26
Mutation (−)	64	17	81
Total	67	40	107

**Table 2:** *EGFR* resistant mutations (p.T790M) in cfDNA and FFPE samples

FFPE	T790M (+)	T790M (−)	Total
cfDNA			
T790M (+)	9	0	9
T790M (−)	11	87	98
Total	20	87	107

**Table 3:** Parameters of CastPCR detecting *EGFR* and *BRAF* mutations in cfDNA#

Parameters	*EGFR*	*BRAF*
Sensitivity	56.4%(31/55)	28.6% (2/7)
Specificity	94.2%(49/52)	93.0% (93/100)
Concordance	74.8%(80/107)	88.8% (95/107)
PPV	91.2% (31/34)	22.2% (2/9)
NPV	67.1% (49/73)	94.9% (93/98)

# Mutation results of FFPE was set the gold standard

PPV: Positive Predictive Value; NPV: Negative Predictive Value.

**Table 4:** Parameters of CastPCR detecting *EGFR* mutations in cfDNA#

Parameters	Del19	L858R	Del19 L858R	T790M
Sensitivity	66.7% (14/21)	45.0% (9/20)	57.5% (23/40)	45% (9/20)
Specificity	96.5% (83/86)	100.0% (87/87)	95.5% (64/67)	100%(87/87)
Concordance	90.7% (97/107)	89.7% (96/107)	81.3% (87/107)	89.7%(96/107)
PPV	82.4% (14/17)	100.9% (9/9)	88.5% (23/26)	100.0%(9/9)
NPV	92.2% (83/90)	88.8% (87/98)	79.0% (64/81)	88.8%(87/98)

#Mutation results of FFPE was set the gold standard

PPV: Positive Predictive Value; NPV: Negative Predictive Value.

### BRAF mutations

In 107 specimens, 8.4% (9/107) were *BRAF* mutated. p.V600E accounted for 33.3% (3/9) and non-p.V600E accounted for 88.9% (8/9). Notably, 4 patients had complex mutations: 1 with p.D594G + p.V600E, 1 with p.G469A + p.D594G + p.V600E and 2 with p.G469A + p.D594G mutations. While in FFPE samples, 6.5% (7/107) patients were identified with *BRAF* mutations. 14.3% (1/7) was p.V600E and 85.7% (6/7) were non-p.V600E. Among the 6 non-p.V600E patients, p.D594G accounted for 83.3%(5/6) and p.G469A accounted for 16.7% (1/10). (Tables 4, 5) Sensitivity, specificity, concordance, PPV and NPV were 28.6% (2/7), 93.0% (93/100), 88.8% (95/107), 22.2% (2/9) and 94.9% (93/98), respectively.

**Table 5:** All the cases harboring *BRAF* mutations

Number	cfDNA	FFPE	Distant Metastasis
1	V600E	V600E	No
2	G469A	G469A	No
8	G469A	No	No
9	G469A	No	Pleura, Bone
10	G469A + D594G	No	Pleura, Lungs, Axillary Nodes
11	G469A + D594G	No	No
12	G469A + D594G + V600E	No	Lungs, Bone
13	D594G + V600E	No	No
14	D594G	No	Bone
3	No	D594G	No
4	No	D594G	No
5	No	D594G	Bone
6	No	D594G	Pleura
7	No	D594G	Pleura, Bone, Lung

## DISCUSSION

As the flourishing development of precision medicine in lung cancer, there is a pressing need for real-time information of clinical specimens, as well as the establishment of a detection platform with convenience, efficiency and economy. With its relative non-invasiveness, high repeatability and real-time dynamic, liquid biopsy is breaking new ground. For the first time, we evaluated the feasibility of CastPCR detecting *EGFR* and *BRAF* mutations in plasma cfDNA of a cohort lung adenocarcinoma patients.

Studies employing different methods detecting *EGFR* sensitive mutations in paired NSCLC tumor tissue and plasma were listed in Table 6. Though Amplification refractory mutation system (ARMS) is extensively adopted to detect tissue *EGFR* mutations in clinical practice, its sensitivity in cfDNA is not stable [34–36]. For denaturing high performance liquid chromatography (DHPLC), its complex detection procedures hinders its clinical promotion [10, 37, 38]. Several targeted or sequencing methods have shown good sensitivity (78.0–81.1%) and concordance (86.0–93.6%) on *EGFR* mutation detection, such as droplet digital PCR (ddPCR) and next-generation sequencing (NGS) [21]. Compared with their prohibitive cost, complex laboratory manipulations and high demand for operational platform, CastPCR is more feasible and economical.

**Table 6:** Studies evaluating cfDNA *EGFR* sensitive mutations in patients with NSCLC

Study	Detection Method	Mutated patients (*n*)	Sensitivity (%) (*n*/total)	Specificity (%) (*n*/total)	Concordance (%) (*n*/total)
Bai et al. [10]	DHPLC	77	81.8 (63/77)	89.5 (137/153)	74.0 (200/230)
Huang et al. [53]	DHPLC	296	63.5 (188/296)	84.6 (445/526)	77.0 (633/822)
Liu et al. [35]	ARMS	40	67.5 (27/40)	100.0 (46/46)	84.9 (73/86)
Kim et al. [54]	PNA-PCR	35	17.1 (6/35)	100.0 (5/5)	27.5 (11/40)
Zhao et al. [55]	Mutant-enriched PCR	45	35.6 (16/45)	95.5 (63/66)	71.2 (79/111)
Wang et al. [36]	ARMS	68	22.1 (15/68)	97.0 (64/66)	59.0 (79/134)
Jing et al. [56]	HRMA	45	66.4 (29/45)	97.3 (73/75)	85.0 (102/120)
Weber et al. [57]	Cobas *EGFR* test	28	60.7 (17/28)	96.4 (162/168)	91.3 (179/196)
Yu et al. [58]	ddPCR	93	61.3 (57/93)	96.7 (118/122)	81.4 (175/215)
Zhu et al. [59]	ddPCR	37	81.1 (30/37)	97.0 (131/135)	93.6 (161/172)

Abbreviations: DHPLC, denaturing high-performance liquid chromatography; ARMS, amplification refractory mutation system; PNA, peptide nucleic acid; PCR, polymerase chain reaction; HRMA, high resolution melting analysis.

*EGFR* T790M plays a key role in selecting the right patient for third-generation *EGFR*-TKI [39]. Up to date, U.S. Food and Drug Administration approved Cobas^®^ as the only assay to detect *EGFR* specific mutations (i.e., exon 19 deletions and exon 21 L858R substitution mutations) in plasma specimens (www.fda.gov). In recent studies that adopted Cobas^®^ testing platform for p. T790M mutation detection, the sensitivity was (51.0%) [40]. In our study, sensitivity detecting p.T790M was 45.0% (9/20). Notably, the specificity and concordance of p.T790M detection was 100.0% (87/87) and 89.7% (96/107), respectively. While in Cobas^®^ the data presented as 77.0% and 61.0%, respectively [40]. Since the PPV and NPV for p.T790M was 100.0% (9/9) and 88.8% (87/98) in our study, for patients who acquire drug resistance to first generation *EGFR*-TKI, plasma detection of *EGFR* p.T790M by CastPCR could be first consideration. For those with cfDNA tested positive, third generation *EGFR*-TKI could be a treatment choice. Similarly, considering the PPV (91.2%, 31/34) and NPV (67.1%, 49/73) of *EGFR* detection with CastPCR in our study, for patients whose tumor tissue is not enough or unavailable, *EGFR* mutation detection with CastPCR in cfDNA could be first choice. For those with *EGFR* mutations in cfDNA, the result may be taken as reference for further clinical medication. While for those detected without *EGFR* mutations in cfDNA, further detection in tumor tissue is recommended. In this way, tumor tissue could be economized to the maximum extent and the risk of repeated percutaneous transthoracic lung biopsy could also be lowered to the maximum extent.

*BRAF* is another important drugable driver mutation in NSCLC. Compared with *EGFR*, the detection of *BRAF* mutation both in tumor tissue and plasma still has a long way to go. To the best of our knowledge, *BRAF* detection of cfDNA in NSCLC hasn't been reported yet. We performed *BRAF* mutation detection in 107 pairs of lung adenocarcinoma plasma cfDNA and matched FFPE DNA samples. CastPCR was applied to detect *BRAF* mutations in tumor tissues and 6.5% (7/107) patients were identified as mutation positive, consistent to the result concluded by NGS [32, 41]. Interestingly, p.V600E accounted for about half *BRAF* mutations in NSCLC in earlier studies [42–46], and recent studies employing NGS technology exhibited that non-p.V600E mutations is the majority (70.0–80.0%) of *BRAF* mutations in NSCLC [32, 41, 47]. In our study, the frequency of non-p.V600E was 85.7% (6/7), similar to that demonstrated by studies using NGS.

In *BRAF* mutation detection of cfDNA with CastPCR, the sensitivity, specificity and concordance was 28.6% (2/7), 93.0% (93/100) and 88.8% (95/107), respectively (Table 3). The low sensitivity may mainly related to the following reasons: Firstly, tumor heterogeneity. In the 12 patients whose mutation conditions were inconsistent, 58.3% (7/12) had distant metastasis. Thus their cfDNA released in plasma could probably carry different gene information compared with primary tumors. Secondly, detection of *BRAF* non-p.V600E mutations in exon 15 of cfDNA needs more investigation. In our study, sensitivity for p.V600E (locates on exon15) and p.G469A (locates on exon11) both reached 100.0%, while none of the five p.D594Gs (locate on exon15) in FFPE samples were detected in their matched plasma. Similarly, Couraud et al. [48] applied NGS to detect mutations of exon 11 and 15 in about 60 tumors and corresponding plasma, and better result was produced for mutations of exon 11 mutations than that of exon 15.

Complex *BRAF* mutations have been reported in different solid tumors. For example, c.1801A>G (p.K601E) + c.1796C>G (p.T599I) in thyroid carcinoma [50], p.T599I + c.1798_1799delinsAA (p.V600K) and c.1798A>T (p.T599T) + p.V600E + c.1803A>T (p.K601N) in melanoma [50, 51]. And we firstly found complex *BRAF* mutations in plasma cfDNA of lung adenocarcinoma: two with p.G469A + p.D594G, one with p.D594G + p.V600E and one with p.G469A + p.D594G + p.V600E. The meaning of these complex mutations still needs to be excavated in the future.

## MATERIALS AND METHODS

### Materials

**Figure 1:**
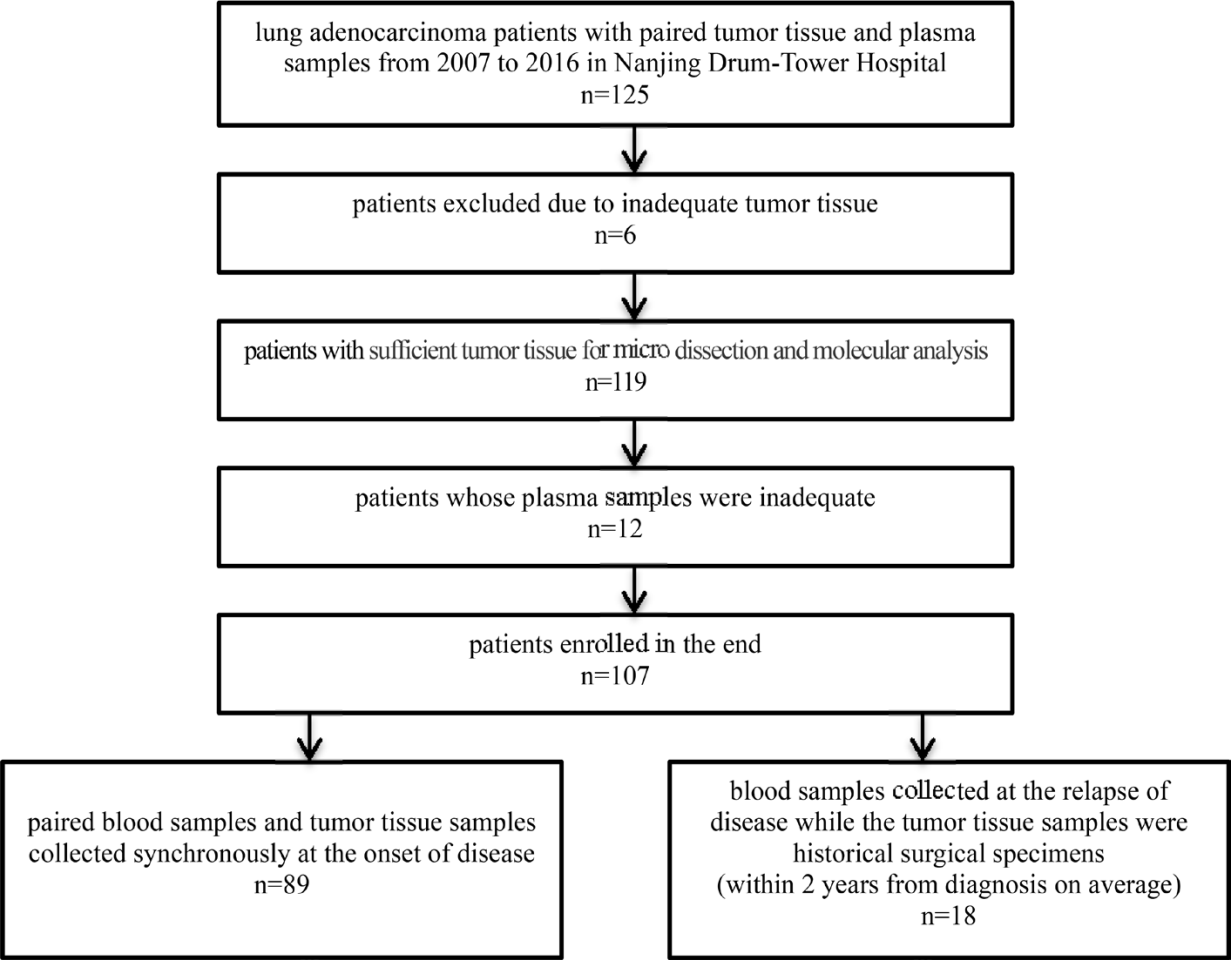
Workflow of patients enrollment

All samples used in this study were obtained from Nanjing Drum-Tower Hospital form 2007 to 2016. 125 lung adenocarcinoma patients were enrolled, and data of 107 paired samples was analyzed in the end (Figure 1). Patients' clinicopathological features are provided in Table 7. 83.2% (89/107) blood samples was collected when patients were diagnosed, together with their paired tumor tissue. 16.8% (18/107) blood samples was collected right after the recurrence, while tumor samples came from the historical surgical specimens (duration of diagnosis to recurrence ranges from 1 to 46 months, medium 18 months), and 61.1% (11/18) of them had experienced chemotherapy after surgery.

**Table 7:** Clinicopathological features of the 107 lung adenocarcinoma patients

Clinicopathological Features	Number of Cases	%
Gender		
Male	60	56.1
Female	47	43.9
Age (years)		
≥ 60 60	53	49.5
< 60	54	50.5
Smoking History		
Smokers#	47	43.9
Non-smokers	60	56.1
Distant Metastasis		
Yes	62	57.9
No	45	42.1
Differentiation Degree		
Well	9	8.4
Medium-Poor	98	91.6
Clinical Stages		
I–III	42	39.3
IV	65	60.7
Tumor Size (cm)*		
≤ 3	16	38.1
≥ 3	26	61.9
Lymph Node Metastasis*		
Yes	31	73.8
No	11	26.2

^#^including 6 former smokers (defined as those had quit smoking ≥ 1 year) [45].

*42 patients that underwent surgical resection were analyzed.

FFPE samples were cut into slices and each slice was 4μm thick. For every FFPE sample, a random slice was performed with the HE staining, then two pathologists identified and marked the tumor tissue through the observation of microscope. 1–2 mL peripheral blood was collected into an EDTA-containing tube, centrifuged at 3000 rpm/min for 15 min at room temperature, then the plasma portion was pipetted carefully, aliquoted to 0.5 mL Eppendorf tubes and stored at −80°C. All patients enrolled in the study provided written informed consent for the use of their resected/biopsied tumor tissue and peripheral blood. The study was approved by the Ethics Committee of Nanjing Drum-Tower Hospital.

### DNA extraction

DNA of FFPE samples was extracted using TIANamp FFPE DNA Kit (Tiangen Biotech, Beijing) according to manufacturer's instructions. For DNA extraction of cfDNA, after comparing the reference Ct value of DNA extracted with MinElute Virus Spin Kit (QIAamp, Germany) and phenol-chloroform method in our preliminary experiment, we adopted the latter method (data not shown). NanoDrop 2000 spectrophotometer (Thermo Fisher Scientific, USA) was used to quantify cfDNA.

### CastPCR

CastPCR technology combines allele specific TaqMan^®^ qPCR with allele-specific blocker (ASB) oligonucleotides that effectively suppress nonspecific amplification from the off-target allele. In our preliminary experiment, we assessed the limit of detection (LOD, minimum percentage of mutant alleles in a wild type background required for reliable mutation detection) of some of the assays with corresponding mutated cell lines. It turned out to be that the sensitivity was consistent to that provided by the manufacturer: 0.1% for *EGFR* c.2235_2249del, p.L858R and *BRAF* p.V600E, 1.0% for *EGFR* p.T790M. Due to unavailable corresponding cell lines, LOD of *EGFR* c.2236_2250del, *BRAF* p.G469A and p.D594G was not assessed. Concentration of extracted DNA was determined by a NanoDrop spectrophotometer (NanoDrop Technologies, Inc., Thermo Fisher Scientific, Wilmington, DE, USA), and adjusted to a concentration of 10 ng/μL. PCR mutation detection assays were then conducted with 2μL of each DNA sample. Every DNA sample was analyzed by CastPCR using the *EGFR*_6223_mu, *EGFR*_6225_mu, *EGFR*_6224_mu, *EGFR*_6240_mu, *BRAF*_460_mu, *BRAF*_467_mu and *BRAF*_460_mu assays for the detection of c.2235_2249del, c.2236_2250del, p.L858R, p.T790M p.G469A, p.D594G and p.V600E, respectively (Life Technologies, USA). The threshold cycle was set as 0.2 according to the handbook of Taqman Mutation Detection Assay by CastPCR. CastPCR was run in a final volume of 10μL in a 96 well plate including 5μL TaqMan^®^ Genotyping Master Mix (2X), 2μL Prepared gDNA sample, 2μL Nuclease-free water and 1μL TaqMan^®^ Mutation Detection Assay(10X) (mutant allele or gene reference assay). Positive control was set for each sample detecting *EGFR* c.2235_2249del, p.T790M, p.L858R and *BRAF* p.V600E. Negative and blank control was set for every assay. Three replicates were set for each assay. PCR were performed on Stratagene MX3000P real-time PCR system (Stratagene, USA). PCR conditions: 95°C for 10 minutes, followed by 5 cycles of 92°C for 15 seconds and 58°C for 1 minute and then 40 cycles of 92°C for 15 seconds and 60°C for 1 minute. According to the handbook of Taqman Mutation Detection Assay by CastPCR, reference Ct value was required between 17 and 27, and samples with a ΔCt of less than 9.96 (9.61 for *EGFR* p.T790M) were considered positive for mutation, where ΔCt = Ct mut - Ct ref. Otherwise the sample was defined as mutation negative. Detection results of tumor tissue was set the gold standard.

### Statistical analysis

Sensitivity, specificity, concordance, PPV and NPV were calculated as follows [52]: Sensitivity = number of true positives/(number of true positives + number of false negatives); Specificity = number of true negatives/ (number of true negatives + number of false positives); Concordance = (number of true positives + number of true negatives) / (number of total cases); PPV = number of true positives/(number of true positives + number of false positives); NPV = number of true negatives/(number of true negatives + number of false negatives).

## CONCLUSIONS

For the first time, we evaluated the possibility of CastPCR detecting *EGFR* and *BRAF* mutations in cfDNA of plasma from 107 lung adenocarcinoma patients. For patients whose tumor tissue is not enough or unavailable, *EGFR* mutation detection with CastPCR in cfDNA could be first choice. For those with *EGFR* mutations in cfDNA, the result may be taken as reference for further clinical medication. While for those detected without *EGFR* mutations in cfDNA, further detection in tumor tissue is recommended. Additionally, CastPCR technology was firstly employed to detect *BRAF* mutations in plasma specimens of lung adenocarcinoma and their matched FFPE samples. We found that for *BRAF* mutation detection, CastPCR is a specific method while its sensitivity, especially for *BRAF* non-p.V600E mutations on exon 15, needs further exploration.
